# Functional SNP allele discovery (fSNPd): an approach to find highly penetrant, environmental-triggered genotypes underlying complex human phenotypes

**DOI:** 10.1186/s12864-017-4325-y

**Published:** 2017-12-04

**Authors:** Kaitlin Stouffer, Michael Nahorski, Pablo Moreno, Nivedita Sarveswaran, David Menon, Michael Lee, C. Geoffrey Woods

**Affiliations:** 1 Cambridge Institute for Medical Research, Wellcome Trust and MRC Building, Addenbrooke’s Hospital campus, Cambridge, UK; 2 Department of Academic Anesthesiology, Addenbrooke’s Hospital Campus, Cambridge, UK

**Keywords:** SNPs, Environmental trigger, Genetic predisposition, Phenotype

## Abstract

**Background:**

Significant human diseases/phenotypes exist which require both an environmental trigger event and a genetic predisposition before the disease/phenotype emerges, e.g. Carbamazepine with the rare SNP allele of rs3909184 causing Stevens Johnson syndrome, and aminoglycosides with rs267606617 causing sensory neural deafness. The underlying genotypes are fully penetrant only when the correct environmental trigger(s) occur, otherwise they are silent and harmless. Such diseases/phenotypes will not appear to have a Mendelian inheritance pattern, unless the environmental trigger is very common (>50% per lifetime). The known causative genotypes are likely to be protein-altering SNPs with dominant/semi-dominant effect. We questioned whether other diseases and phenotypes could have a similar aetiology.

**Methods:**

We wrote the fSNPd program to analyse multiple exomes from a test cohort simultaneously with the purpose of identifying SNP alleles at a significantly different frequency to that of the general population. fSNPd was tested on trial cohorts, iteratively improved, and modelled for performance against an idealised association study under mutliple parameters. We also assessed the seqeuncing depath of all human exons to determine which were sufficiently well sequenced in an exome to be sued by fSNPd - by assessing forty exomes base by base.

**Results:**

We describe a simple methodology for the detection of SNPs capable of causing a phenotype triggered by an environmental event. This uses cohorts of relatively small size (30–100 individuals) with the phenotype being investigated, their exomes, and thence seeks SNP allele frequencies significantly different from expected to identify potentially clinically important, protein altering SNP alleles. The strengths and weaknesses of this approach for discovering significant genetic causes of human disease are comparable to Mendelian disease mutation detection and Association Studies.

**Conclusions:**

The fSNPd methodology is another approach, and has potentially significant advantage over Association studies in needing far fewer individuals, to detect genes involved in the pathogenesis of a diseases/phenotypes. Furthermore, the SNP alleles identified alter amino acids, potentially making it easier to devise functional assays of protein function to determine pathogenicity.

**Electronic supplementary material:**

The online version of this article (10.1186/s12864-017-4325-y) contains supplementary material, which is available to authorized users.

## Background

**Fig. 1:**
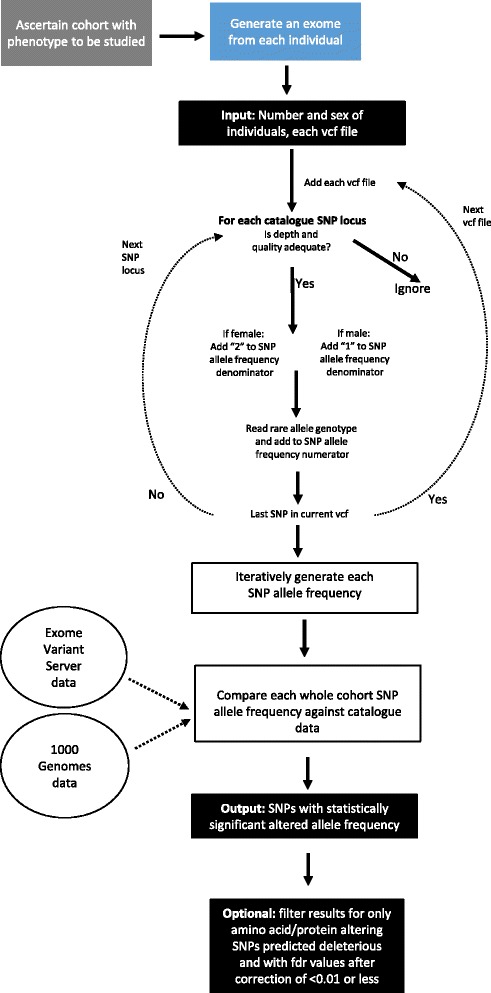
Diagram of the methodological steps of the input, internal processes and output of the “functional single nucleotide polymorphism discovery” (fSNPd) program. Legend: The first step is the ascertainment of a cohort of individuals with the phenotype to be studies; blue box. The second step is to perform exomes (with the same capture and sequencing methods) on a number of the cohort individuals; blue box. The top-most black box shows the input to fSNPd; number of individuals, number of males and females, and each individuals vcf (and if required, bam + bam.bai) files). Entering the number of males and females allows fSNP to internally check that it has assigned sex correctly to each sample, so that SNPs on the X chromosome have correct allele frequencies calculated. A second lower-most black box shows the output of fSNPd, the list of SNPs where the allele frequency is statistically altered compared to the 1000 Genomes or Exome variant Server European library data. Between the black boxes are shown the processes performed within fSNPd

Molecular genetic approaches have proved extremely powerful to discover and dissect the genetic components underlying many human diseases. Clear examples are: the discovery of highly penetrant, but often very rare, pathogenic mutations causing Mendelian diseases [1, 2]; Association Studies using single nucleotide polymorphisms (SNPs) with common rare allele frequencies able to discover haplotypes causing small phenotypic effects, which have revealed many disease processes [3–5] and in cancer where somatic mutation detection *en masse* are delineating hitherto unrecognized tumor types and leading to personalized therapies [6, 7]. We describe here a further genetic approach, functional single nucleotide polymorphism discovery (fSNPd) (summarized in Fig. 1). This was designed to detect genetic components of clinically important disorders that require an environmental trigger to occur, e.g. severe drug reactions, pain syndromes occurring after an injury, and susceptibility to infection.

fSNPd relies upon SNPs, which are widespread and frequent variations found in the genomic sequence of individuals of a species. fSNPd does examine autosomal and sex-chromosome SNPs. The term SNP has, by common usage, come to also include small insertions and deletions (INDELS) in genomic DNA. SNPs have a widespread distribution throughout the human genome [8]. For each SNP, the first allele is almost always the commoner allele, but the rarer second allele can potentially alter the canonical protein sequence or/and protein expression. The majority of SNP second/rare alleles are considered harmless, or at least are not known to exert a phenotypic effect. However, it is known that some of these usually “silent” SNP second alleles do cause a phenotype when combined with a specific environment trigger, e.g. SNPs rs3909184 and rs2844682 (the genotype HLA-B*1502) which are phenotypically silent until after Carbamazepine absorption when Stevens Johnson syndrome (Toxic epidermal necrolysis) occurs, HIV progression and G allele of rs1799987, and mitochondrial genome SNPs rs267606617 (m.1555A > G) and rs267606618 (m.1095 T > C) causing sensory-neural deafness after amino-glycoside administration [9–11].

We hypothesised that other SNPs could also cause environmental triggered diseases and phenotypes. We have developed a simple methodology for their discovery using small cohorts and exomes which we report here.

## Results

The “exome” we used contained 19,214 genes (which were fully or partially sequenced), and approximately 121,000 SNPs where rare alleles were called (which varied between individual’s exomes), and of which 20% were protein changing.

We compared these allele frequencies against “control” allele frequencies obtained from population database catalogues. Firstly, we used the data in the European subset of the 1000G for SNP allele frequencies. And secondly, and separately, the SNP allele frequency data from the EVS [12, 13]. Where there was data in both catalogues, most SNP allele frequencies were similar (<5% difference in allele frequency), but for a minority (17%) there was a potentially significant difference (>5% difference in allele frequency). There were a number of reasons we could identify for this discrepancy, including the fact that some SNPs had more than two alleles in EVS and our approach only enabled us to record the lowest frequency SNP allele - so EVS data for these SNPs rare alleles was incorrect. Other discordances arose from mis-allocation of “reads” to an incorrect gene (e.g. potassium channel genes), or from real differences in the dataset results due to factors such as ethnic differences (e.g. MC1R allele frequencies). Thus, the utility of producing two separate sets of results, one using the European subset of the 1000 Genomes project for normal/control population SNP allele frequencies and the other using Exome Variant Server (EVS) for these frequencies, was to increase the chances of finding real significant results but at the cost of an increased number of false negative and false positive results.

The number of SNPs that could be accurately sequenced in our exomes was determined, and the results are detailed in Additional file 1: Table S1 which gives the results per gene, and per exon of each gene. For satisfactory coverage we used a cutoff of 20 fold coverage, as less is regarded as insufficient to reliably detect heterozygosity. There were data for 19,214 genes. 82% (16,023) genes had an average coverage >19 reads, see Additional file 1: Figure. Our data analysis shows that SNP allele frequency determination is incomplete, but mostly predictably so between tested exomes.

To assess the performance of fSNPd for allele frequency determination we analysed 100 SNPs, chosen from two study cohorts, where allele frequencies were found to be statistically altered and checked the results by use of the Integrated Genome Viewer (IGV [14]); all frequencies were accurate to 1%. We also checked seven of the 100 SNPs by Sanger sequencing of all of the respective cohorts individuals - and found allele frequency results accurate in six, and for one SNP to within 1%. The cause for the difference was that one of the samples analysed for the SNP had a read depth < 20, so was not used by fSNPd in calculating the allele frequency, whereas it was able to be successfully Sanger sequenced and the alleles scored.

We performed fSNPd simulations, assuming that a rare SNP allele was fully penetrant when present (unless otherwise stated), see Additional file 1: Table S2. For a cohort of 100, and a SNP rare allele frequency in the normal population of 0.01 – fSNPd detected the SNP rare disease causing allele when there were one to eight SNPs all capable of causing the investigated phenotype; an Association Study approach detected none with Chi squared *p* value of <10^−8^. If we made the rare allele frequency in the general population 0.005 then fSNPd detected the rare SNP alleles if there were one to ten causative SNPs. If there were only 30 subjects in the cohort, or if we simulated half the cases of a cohort of 100 were non-genetic, or if the SNP rare allele was only penetrant in 50% of cases, then fSNPd could detect the SNP alleles if there were one to four causative SNPs. And, if our cohort had 3000 individuals Association Studies would detected the SNP alleles when there was one to five causative SNPs, and fSNPd one to ten causative SNPs.

Finally we illustrate the potential use of fSNPd by re-examining two of the known examples of human diseases/phenotypes which require both an environmental trigger event and a genetic predisposition before the disease/phenotype emerges. Firstly, 44 patients were reported who had Stevens–Johnson syndrome after taking carbamazepine, all had the G allele of the HLA-B SNP rs3909184, where the allele frequency in the general population 8% [9]; this result would have been detected by fSNPd with a corrected *p* value of <10^−8^. Secondly, deafness caused by aminoglycoside antibiotics found to be due to the mitochondrial genome SNP rs267606617 (m.1555A > G), would not have been detected by the fSNPd method, as the mitochondrial genome is not included in an “exome”, nor was the cohort size of six individuals sufficient [10]. Thirdly, human immunodeficiency virus infection progression and rs1799987 in the promotor of the CCR5 gene where the G allele is protective and has a prevalence of 45% in the general population and 64% in those with longer survival; this would have been detected by fSNPd with a corrected *p* value of <10^−6^.

## Discussion

A number of clear examples are known of disorders which have both an essential environmental and an essential genetic component, and for some of these the etiology is binary; a single environmental trigger and a single genotype are both required. However, it is unclear how many diseases/disorders/phenotypes could have this joint and equal environmental – genetic architecture. Doubtless this architecture will be a simplification. As a consequence we have concentrated on the situation of a single environmental trigger (hence allowing easy cohort ascertainment) and potentially a number of different, but each highly penetrant, genetic changes (which could be in the same or different genes). Adverse drug reactions are an example of this situation, particularly idiosyncratic Type B reactions [15]. Some of the genotypes underlying these reactions have been discovered, but many have not, e.g. glucose-6-phosphate dehydrogenase deficiency (multiple mutations in one gene, some common enough to be known SNPs) who are at significant risk of developing acute hemolytic anemia after sulphonamide administration, and cholinesterase deficiency which is asymptomatic unless the individual is given the anesthetic agent suxamethonium which causes severe prolonged muscle relaxation. There is a need for greater consideration of this situation; the co-occurrence in an individual of the environmental trigger and the genetic change/genotype leads to a significant (>.5) chance of a resulting human phenotype.

fSNPd has the potential to detect diseases and phenotypes that require both an environmental trigger event(s), and the inheritance of a specific susceptible genetic background. The genetic causes of the phenotypes which fSNPd is designed to detect are assumed to be complex: environmental triggers that may be multiple and involve (at least) age, sex and disease state; phenotype penetrance may be incomplete (as for many dominant Mendelian conditions); and multiple independently-penetrant SNP alleles could each cause a clinically indistinguishable phenotype. The SNP alleles that can be discovered by fSNPd would be expected to range from relatively common (<50%), to very rare (<0.1%).

Our comparison of fSNPd to Mendelian gene discovery and Association Studies (Table 1) shows that fSNPd can use far smaller disease/phenotype cohorts compared to Association studies (10s to 100 s versus 100 s to 10,000 s), see Additional file 1: Table S2. As a consequence the lesser cost of association studies per cohort individual is offset against the greater current cost of an exome. Such smaller fSNPd cohorts are easier to collect, and to make ethnically or geographically restricted. As with Association studies, the smaller the cohort in fSNPd the greater the chance of false positive and false negatives for commoner alleles, and false negatives for rarer alleles [16].

**Table 1:** A comparison of the methodologies, strengths and weaknesses of Mendelian gene discovery, Association studies and fSNPd

	Mendelian gene discovery	Genome Wide Association studies	fSNPd
Minimum number of affected individuals/families	2 to 10 families	Typically >2000	30–200 individuals
Scope	Usually exome	Genome	Exome
Approach	Usually candidate	Non-candidate	Non-candidate
Proof	If linkage *p* < 0.05 +/− additional proofs	Conventional threshold is *p* < 5 × 10^−8^	*p* < 0.01 after correction for SNPs assessed
Effect on phenotype	Fully penetrant for recessive and X-linked; dominant penetrance 0.33 to 1.0	Usually very small, e.g. odds ratio < 1.33 for a risk SNP.	Non-penetrant before environmental triggers; presumed 0.5–1.0 penetrance after trigger.
Approximate cost to perform	£10,000–£100,000	£300,000–£8,000,000	£15,000–£50,000
Number of phenotype-associated genes identified	1	>1	>1
Are changes discovered easily functionally assessed	Yes	Usually No	Yes
Ability to cope with non-genetic cases	Poor	Moderate	Good

## Conclusions

We suggest that the fSNPd approach should be considered when seeking the highly penetrant genetic components for apparently sporadic phenotypes which can be triggered by environmental events. As the common causative SNP alleles will be discovered in one experiment, and each SNPs is within a single gene, and the SNP alleles cause protein changes, then fSNPd has the potential to facilitate the rapid discovery of underlying pathophysiology.

## Methods

Our aim was to find gene changes with fully penetrant effects that would cause pain phenotypes. We designed our approach based on three tenets. Firstly, the SNP alleles that would individually cause fully penetrant (and detectable) effects were most likely to be exonic and protein changing (mis-sense, non-sense, indels, or splicing). Secondly, an exome would detect the majority of these SNPs. Thirdly, smaller cohorts are easier to collect, phenotype and curate.

We wrote the fSNPd program to analyse multiple exomes simultaneously with the purpose of identifying common SNP alleles that exist in a population of interest at a significantly different frequency than that of the general population. To do this, the program first establishes a catalogue of common SNPs based on an existing cohort; we used the 1000 Genome Project or the Exome Variant Server (EVS) as both were available at the time to download. Confounding variables can be limited by choosing a subset of such a cohort based on ethnicity/location to match the demographics of the population of interest.

In our experiments, we tested the European subset of the 1000 Genome Project (the UK subset being unfortunately too small for this purpose – about 100 individuals) and the EVS, and considered SNPs that differed from at least one of these at a significant level to be relevant. The 1000 Genome Project had the advantage of being a better ethnic match for our cohort (predominantly white Caucasian), while the EVS had data from a greater number of individuals (approximately 20,000 individuals). In the final program fSNPd produces results for both.

The program calculates rare allele frequencies of SNPs in the population of interest. Individuals’ data is handled in vcf format (post variant-calling) versus raw sequencing reads, so that each locus is assigned two possible alleles. Population allele frequencies are determined by tallying the counts of each allele at each SNP locus across all of the individuals in the population of interest and dividing by the total number of presumed alleles at each locus (two per individual for loci on autosomal chromosomes and one (male) or two (female) per individual for those on sex chromosomes).

Finally, allele frequencies per each SNP in the starting catalogue are compared between the population of interest and the general population using a two-tailed chi-squared test without Yates’ correction. SNPs with significantly higher or lower allele frequencies in the population of interest are identified. Significance is characterized by frequencies that differ by a *p*-value of 2% or more, with Bonferroni and false discovery rate (FDR) corrections applied, according to the total number of SNPs compared (the number in the initial catalogue from the existing cohort) [17].

Additionally, for SNPs that do not exist in particular individuals in the population of interest, raw sequencing data can be accessed to confirm coverage of the area. If no coverage exists, fSNPd can calculate upper and lower bounds of allele frequencies and subsequent *p*-values.

The data input for the final fSNPd program are each individual’s vcf file, and the number of males and females. As an option the fSNPd program can check the actual read depth and quality of each SNP called, if this option is selected, each cohort individuals’ bam and bam.bai files are required. See Additional file 1: Supplementary data for fSNPd set up, and URL’s for downloading programs.

We wanted to determine how many SNPs were accurately sequenced in an exome, and so analysed 40 exomes by fSNPd to determine base by base and exon by exon coverage. This analysis sought to determine whether particular genes or exons of genes would be predictably included in, or missed from, our results.

We assess the performance of fSNPd for allele frequency determination by analysis of two patient cohorts (one of 116 individuals, the other of 34). From the results we selected a sample of SNPs from each where allele frequencies were found to be statistically altered and checked the results “by hand” by use of the Integrated Genome Viewer and by Sanger sequencing of all of the cohort individuals.

After generating results for all SNPs encompassed by an exome we chose to examine only those SNPs that would unequivocally alter proteins (nonsense mutations, start and stop codon mutations, canonical splice site mutations, and missense mutations predicted to be potentially pathogenic). This was for two reasons; such mutations are easier to assess by bio-informatics analysis, and are more easily amenable to functional testing to determine their pathogenicity. Others, however, may seek to examine all of the SNPs identified by the pipeline.

We simulated the performance of fSNPd under a variety of conditions, see Additional file 1: Table S2.

fSNPd is freely downloadable, instructions are in the Supplement [18].

## Additional file

Additional file 1: Use of fSNPd and the Supplementary Figure. This firstly documents the instructions for the installation of fSNPd, and its use. Secondly, it contains the Supplemental Figure entitled: Average read depth per base of genes in a test cohort of 40 individuals’ exomes - data derived from Supplemental **Table S1.** This shows a graph of calculate the average read coverage by all genes, and the legend details the methodology used. References used are given after the legend. Read number statistics of average depth of sequence reads for each exon of each gene included in the exomes of forty individuals. This is a large spreadsheet giving the detailed analysis of read depth achieved. Results and statistics are given for each gene and exon of each gene. Thus, the coverage of an exome of any desired exon can be determined. Simulations of the performance of fSNPd compared to an Association study approach in a variety of scenarios. Simulations were performed assuming that a rare SNP allele was fully penetrant when present (unless otherwise stated). For each Simulation SNP-1 is a common SNP (such as are used in Association studies) with allele A frequency = allele B frequency = 0.5 and SNP-2 (analysed in fSNPd) has the rare allele A frequency = .01 and the common allele B frequency = .99. The SNP-2 allele A is disease associated and always on a SNP-1 allele A background. Simulation are of: the number of SNPs causing a phenotype; different SNP-2 rare allele frequencies; if half of cases are non-genetic or rare SNPs allele penetrance 50%; and of using a cohort size of 3000, rather than 100 as used for all other simulations. (ZIP 8041 kb)

## Abbreviations

EVS: Exome Variant Server
FDR: False discovery rate
fSNPd: Functional single nucleotide polymorphism discovery
INDELS: Small insertions and deletions in genomic DNA
SNPs: Single nucleotide polymorphisms
