# Supplementary material for: Functional SNP allele discovery (fSNPd): an approach to find highly penetrant, environmental-triggered genotypes underlying complex human phenotypes
Source: BMC Genomics. 2017 Dec 4;18:944. doi: 10.1186/s12864-017-4325-y (PMC5716007; doi:10.1186/s12864-017-4325-y)
Supplement: Additional file 1: — Use of fSNPd and the Supplementary Figure. This firstly documents the instructions for the installation of fSNPd, and its use. Secondly, it contains the Supplemental Figure entitled: Average read depth per base of genes in a test cohort of 40 individuals’ exomes - data derived from Supplemental Table S1. This shows a graph of calculate the average read coverage by all genes, and the legend details the methodology used. References used are given after the legend. Read number statistics of average depth of sequence reads for each exon of each gene included in the exomes of forty individuals. This is a large spreadsheet giving the detailed analysis of read depth achieved. Results and statistics are given for each gene and exon of each gene. Thus, the coverage of an exome of any desired exon can be determined. Simulations of the performance of fSNPd compared to an Association study approach in a variety of scenarios. Simulations were performed assuming that a rare SNP allele was fully penetrant when present (unless otherwise stated). For each Simulation SNP-1 is a common SNP (such as are used in Association studies) with allele A frequency = allele B frequency = 0.5 and SNP-2 (analysed in fSNPd) has the rare allele A frequency = .01 and the common allele B frequency = .99. The SNP-2 allele A is disease associated and always on a SNP-1 allele A background. Simulation are of: the number of SNPs causing a phenotype; different SNP-2 rare allele frequencies; if half of cases are non-genetic or rare SNPs allele penetrance 50%; and of using a cohort size of 3000, rather than 100 as used for all other simulations. (ZIP 8041 kb) [file 12864_2017_4325_MOESM1_ESM.zip › fSNPd BMC Genomics Supplementary Material.pdf]

Supplemental data

Using fSNPd

<u>fSBPd set up</u>
fSNPd runs in a LINUX environment with a SLURM controller, if you use a different controller then the files will have to be altered accordingly.
The following programs are necessary - if not present install using conda install; python-2.7, virtualenv, pysam, htslib, bzip2, samtools, bedtools, data.table, optparse, and r.
Make yourself a master folder, and then subfolders named "Annotated_VCFs", "Bam_Files", and "results".
Download all files in https://github.com/pcm32/woods-variation-wrappers into your master folder.
Rewrite the locations of your programs and folders in "settings.sh".

<u>Running fSNPd</u>
To run an fSNPd analysis copy the vcf results file of the exome of each individual in the cohort for analysis into the folder "Annotated_VCFs", using a program such as WinSCP.
If you require them, copy the bam and bam.bai files of the exome of each individual in the cohort for analysis into the folder "Bam_Files".
Navigate to your folder in a command line program, such as PuTTY.
Type "bash toughMums.sh", and the program will guide you through entering data and the options you can select or leave as default.
Run the program, and you will be given a unique identifier for the error, report and results files generated during fSNPd running.
Results will appear in your "results" folder as two excel spreadsheet. One contains SNP data, the second contains other nucleotide changes unrecognised as SNPs found – which will be generally very rare SNPs.
Optional: we then filter the results files. We eliminate all intronic and synonymous SNPs, and those mis-sense SNP alleles not predicted to be pathogenic by SIFT. Potential splicing mutations may require hand curation if they don't involve the canonical splice acceptor AG and splice donor GT nucleotides, before being eliminated. We usually start with "1000 Genomes European results" and ignore all SNP results with an fdr <0.001, although more stringent p values could be used these run the risk of eliminating very rare allele frequencies that have increased.

Figure

<u>Supplemental Figure</u>
Average read depth per base of genes in a test cohort of 40 individuals' exomes, data derived from Supplemental Table S1.

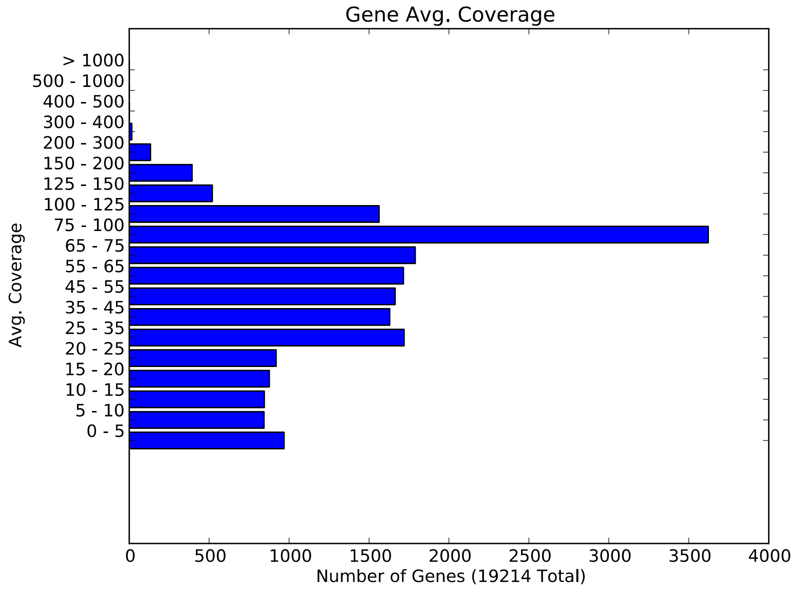

To calculate the average coverage (# reads/base) by exon and by gene, we developed a small program which utilized SAMTools' PySam library[1] to extract the number of reads per base from the BAM files for 40 exon sequences (generated in the same batch) from individuals with a range of Mendelian disorders. We recorded the total reads only for those bases included in coding regions of exons, according to the RefSeq gene coordinates in the UCSC databases[2,3]. For each individual, we calculated average coverage per exon and per gene by summing the total reads per base over each coordinate in coding region(s) and dividing by the total number of these coordinates in each exon or gene, respectively. To calculate averages across the cohort, we summed calculated averages per exon and per gene and divided by the total number of individuals (40).

1. Heger A, Belgrad TG, Goodson M, Goodstad L, Jacobs K. pysam: samtools interface for python. www.cgat.org/~andreas/documentation/pysam/contents.html#.
2. Pruitt KD, Tatusova T, Maglott DR. NCBI Reference Sequence (RefSeq): a curated non-redundant sequence database of genomes, transcripts and proteins. Nucleic Acids Research 2005; 33(suppl 1): D501-D504.
3. Karolchik D, Barber GP, Casper J, Clawson H, Cline MS, Diekhans M, et al. The UCSC Genome Browser database: 2014 update. Nucleic Acids Research 2014; 42(D1): D764-D770.

Tables (as separate Supplementary files)

Supplemental Table S1
Title: Base coverage in 40 exomes

Legend: The results are given for each known gene and exon of known genes, and also for only those genes encoding proteins. The analysis is the read depth per base for each exon and gene. Further details are included within the spreadsheet.

Supplemental Table S2
Title: Simulations of the performance of fSNPd and Association studies.

Legend: The results of simulations are shown allowing the potential performance of fSNPd to be assessed in different situations. For each set of simulations SNP-1 has two common alleles (A and B) both at .5 frequency. SNP-2 has a rare allele (A) that is causes disease combined with an environmental trigger. The SNP-2 allele A always occurs on the background of SNP1- allele A. The significance by Chi squared analysis is given, as is a typical Bonferroni correction of 100,000.

Simulation 1 has SNP-2 rare allele frequency set at 0.01, the SNP-2 allele A was fully penetrant, and there were 100 individuals in the phenotype cohort. The number of different SNP which have disease-causing rare alleles is increased from one to ten. Or by corollary, it looks at disease causing alleles with a population frequency of 0.01 that can be detected with a disease cohort frequency varying from 0.5 to 0.05. The result shows that detecting a rare disease causing SNP allele would occur in all situations tested. However, seeking a change in the common SNP allele is only effective when there are one or two disease causing rare SNPs.

Simulation 2 uses the same parameters except that only 30 individuals are in the phenotype cohort. fSNPd detected the SNP-2 rare allele when the number of disease causing SNPs was one to six (allele frequency in phenotype cohort 0.5 to 0.83). The change in the common SNP allele was not detected in any of the simulations.

Simulation 3 used the same parameters as but assumed half of the cohort had a non-genetic cause of their phenotype. fSNPd detected the SNP-2 rare allele when the number of disease causing SNPs was one to five (allele frequency in phenotype cohort 0.5 to 0.1). The change in the common SNP allele was not detected in any of the simulations. The same results are produced if the disease associated SNP-2 allele A penetrance was only 50%.

Simulation 4 looked at the effect of using a 0.005 allele frequency for the disease causing SNP-2 allele A. Other parameters were as in Simulation one. fSNPd would detect the disease causing allele when the number of disease causing SNPs was one to six (allele frequency in phenotype cohort 0.5 to 0.05), but failed to do so when there were 50 SNPs. The change in the common SNP allele was only detected when there are one or two disease causing rare SNPs.

Simulation 5 compared fSNPd to results that might be produced from an association study of 3000 individuals. One to ten disease-associated NSPS were modelled (allele frequencies from 0.5 to 0.05 in the phenotype cohort). Otherwise parameters are as in Simulation one. When there was between one and six disease causing SNPs (or disease associated SNP alleles with a frequency of 0.5 to 0.83) a statistically significant increase was detected using the common SNP. However, fSNPd potentially detected all of the rare disease associated SNPs.
